# Reproducibility and agreement between three positions for handgrip assessment

**DOI:** 10.1038/s41598-021-92296-8

**Published:** 2021-06-18

**Authors:** Olga-Cecilia Vargas-Pinilla, Eliana-Isabel Rodríguez-Grande

**Affiliations:** Physical Therapy Program, Rehabilitation Science Research Group, School of Medicine and Health Sciences, Universidad del Rosario, Carrera 24 # 63C – 69, 111221 Bogotá, Colombia

**Keywords:** Health care, Medical research

## Abstract

The protocol established for taking hand grip dynamometry measurements determines that the patient must be in a sitting position. This protocol cannot be applied due to the patient’s conditions in some cases, such as abdominal surgery, musculoskeletal spine or hip injuries. The purpose was to determine the reproducibility and level of agreement between the Handgrip dynamometry in supine position with the elbow flexed or extended, and the one measured in the sitting position, the design was a descriptive cross-sectional study. The population were young apparently healthy between 18 and 30 years of age (N = 201). Handgrip measurement was performed on both upper limbs in a sitting position with a flexed elbow, a supine position with a flexed elbow, and supine position with the elbow extended. Reproducibility was nearly perfect in all positions (ICC 0.95–0.97). Regarding the level of agreement for the comparison between sitting and supine positions with a flexed elbow, an average difference of − 0.406. For supine position with an extended elbow and supine position with a flexed elbow, the average difference was − 1.479. Considering the results, clinicians or researchers can choose any of the positions evaluated herein and obtain reliable results as long as the standardization process is followed.

## Introduction

Muscle strength is a functional physical quality that is essential for the fulfillment of daily activities performed by human beings. It can be defined as the ability of a muscle group to develop maximum contractile strength against resistance in a single contraction^1,2^.

The gripping strength employed by the thumb and the four fingers against a contact surface is known as Hand Strength (HS)^2^. It is measured using a hand dynamometer and is the gold standard for the assessment of overall muscle strength due to its association with the strength of lower limbs, muscle mass and muscular cross-sectional area. Zengin et al., evaluated in three hundred and one men the grip strength using a Jamar dynamometer, and the cross-sectional muscle area with a computed tomography at 50% of the radius and 38% and 66% of the tibia in the non-dominant side. They found a positive relationship between grip strength z‐score and cortical bone mineral content, cross‐sectional area and cross-sectional muscle area^3^. Strandkvist et al.^4^ measured the hand grip strength and lower limb strength of forty-five individuals over 70 years of age. They found that lower limb strength explained 74.4% of the variance in hand grip strength; so they highlight that lower limb strength and hand grip strength were strongly associated and support that hand grip strength is a valid method to estimate lower limb strength among older adults.

HS is a crucial indicator that has major clinical implications^5,6^. It has a close relationship with lean mass, making it a determining factor of the overall functional integrity of patients^7–9^. In addition to being an indicator of patient functionality, it has been reported as an index that is associated with premature death, recovery time, and post-surgery functionality^10–13^. Hand strength measured with a hand dynamometer in a pre-surgical evaluation may be used for determining the risk of post-surgical complications and a longer hospital stay^13^. A low HS level has been associated with a higher level of disability and other health-related complications in the elderly^10,14,15^.

Hand Grip Dynamometry (HGD) is one of the most widely used and validated methods for the evaluation of HS, and it is considered an objective measurement for tracking changes in muscle strength^16,17^. Presently, this is the simplest method for evaluating maximum voluntary muscle function as it is an exploratory method that is quick and easy to perform, which usually estimates the overall muscle strength of the body with high reliability^18^.

Considering the importance of this indicator, the measurement process must be standardized and controlled in each of its phases for achieving valid and reliable results. When performing measurements using hand grip dynamometer, the application protocol should be standardized since variables such as hand dominance and the position assumed by the person being evaluated influence the variability of the result^18^. The protocol established by the American Society of Hand Therapists for taking measurements of HGD determines that the patient must be seated with a neutral shoulder position, the elbow flexed at 90°, and the forearm and wrist in neutral positions^19^. However, in people, such as those with abdominal or pelvic surgery, injuries of the musculoskeletal spine or hip, or the presence of a monitoring equipment, who cannot adopt the sitting posture, this protocol of HGD cannot be applied.

Few studies have evaluated the reproducibility of HGD results measured at different positions. Hillman et al., evaluated the HGD in a healthy population in three different positions with the elbow flexed: supine, sitting with support, and sitting without support. When the results were compared, no statistically significant difference was noted between the grip strength of the dominant hand measured in the three positions^6^.

However, this study did not include the elbow extended position, which would have been important, considering that some inpatients, even in supine posture, may not have the necessary strength to keep the elbow flexed. Therefore, the purpose of this study was to determine the reproducibility and level of agreement between the HGD measured in the supine position with the elbow flexed or extended, and the one measured in the sitting position for determining if these postures can be interchangeable.

## Methods

### Participants

Considering that the objective of the study was to evaluate the psychometric properties of HGD and not to evaluate the HS of people in clinical conditions with limited mobility, a potentially healthy population was included. This study used a convenience sampling that included apparently healthy students from the School of Medicine and Health Sciences from a university, who were young adults between 18 and 30 years of age. After receiving information regarding the study, all participants formalized their participation by signing an informed consent form in accordance with the guidelines present in the Declaration of Helsinki. Participants with a musculoskeletal injury of the upper limbs or pain at the time of testing were excluded. The sample size calculation for two-tailed confidence intervals, with three measurements per participant, an expected correlation between 0.8 and 0.9, an accuracy of 95%, a power of 90%, yielded a size of 180 participants. All analyses were performed in Stata 14 software^20^.

### Study design

This was a descriptive cross-sectional study. A Takei GRIP-A dynamometer was used, which was calibrated prior to each test. The HGD was performed on both upper limbs in a sitting and supine posture with flexed elbow and a supine posture with extended elbow. All tests were performed in the biomechanics laboratory of the School of Medicine and Health Sciences from January to July 2019.

### Study protocol

The sampling method was by convenience. Participants were informed of the measurement protocol; subsequently, information on age, sex, and hand dominance was collected. Before beginning to perform the HGD, the evaluator demonstrated the test in each of the three positions. Three measurements were taken for each upper limb in each of the three positions and each effort maintained between 3 and 5 s.

The evaluator used standardized verbal commands during measurements in order to achieve maximum effort from each participant. The evaluator was a student in her last semester of physiotherapy who was trained for the application of the test and followed a standardized procedure.

The order of the positions for each participant was determined by random numbers and the measurements started with their dominant hand. A resting period of 2 min was allowed between each test and the participant was unable to see the results of each test.

### Measurement positions

Sitting: the participant sits comfortably in a firm chair with a back and no armrests. The shoulder is in a neutral position, the elbow is flexed at 90° and attached to the trunk, and the forearm and wrist are in neutral positions.

Supine position with flexed elbow: the participant is in a supine position with a neutral shoulder position, the elbow is flexed at 90° and attached to the trunk, and the forearm and wrist are in neutral positions.

Supine position with extended elbow: the participant is in a supine position with the shoulder in a neutral position, the elbow is extended at 0° and attached to the trunk, the forearm is in a supine position and the wrist is semi-flexed.

### Statistical analysis

Descriptive statistics were applied to the variables collected in the study using the measurements of central tendency (average and median) and dispersion (standard deviation and interquartile range), depending on the distribution of the variables. Reproducibility was determined by Intraclass Correlation Coefficient (ICC 2,1). The ICC results were interpreted according to the Landis and Koch classification as follows: the values of 0.81–1.00 indicated almost perfect agreement; the values of 0.61–0.80 indicated considerable agreement; the values of 0.42–0.60 indicated moderate agreement; the values of 0.21–0.40 indicated fair agreement; the values of 0.00–0.20 indicated low agreement; and the values < 0 indicated poor agreement. Confidence intervals were calculated with a 95% confidence level for concordance^21^.

For determining the variation of the pounds of strength between one posture and another, the level of agreement was calculated using the Bland and Altman’s graphic analysis. All analyses were performed using Stata 14 statistical software^20^.

### Ethical considerations

This study was approved by the Research Ethics Committee of the Fundación Cardioinfantil de Colombia, under registration number 05-2018, and it followed all the national and international standards that apply to human research in accordance with the guidelines present in Declaration of Helsinki. The students voluntarily agreed to participate by signing an informed consent form. To maintain confidentiality, we assigned a code to the evaluators and to those being evaluated (instructors and students); we used these codes in the subsequent evaluation tools and data analysis.

## Results

Hundred sixty-six women and thirty-five men participated in the study; 92% of the participants were right-handed. The volunteers were students from The School of Medicine and Health Sciences. None of the participants reported musculoskeletal disorders nor were any missing data in the intra-evaluation reproducibility measurements collected (Table 1).

**Table 1:** Characteristics of the participants.

Variable	n 201 (%)
**Gender**	
Male	35 (17.4%)
Female	166 (82.59%)
**Dominance**	
Right	185 (92.04%)
Left	16 (7.96%)
Age	20.3 ± 2.6^b^
**Right upper limb muscle strength**	
Supine with flexed elbow	21.67 (18.67, 24.6)^a^
Supine with extended elbow	23 (19.33,26.6)^a^
Sitting	21.33 (19,2, 25)^a^
**Left upper limb muscle strength**	
Supine with flexed elbow	20.33 (18,2, 24)^a^
Supine with extended elbow	21.33 (18.33, 25.6)^a^
Sitting	20 (17.33,24.33)^a^

^a^Data are presented as median (quartile 25, quartile 75).

^b^mean ± standard deviation.

Considering that three measurements were taken in each position, the difference between the highest measurement achieved in the three attempts and the average value of the three attempts was compared. No statistically significant differences were noted in this comparison in any of the positions, so the results are reported using the average value of the three measurements.

**Figure 1:**
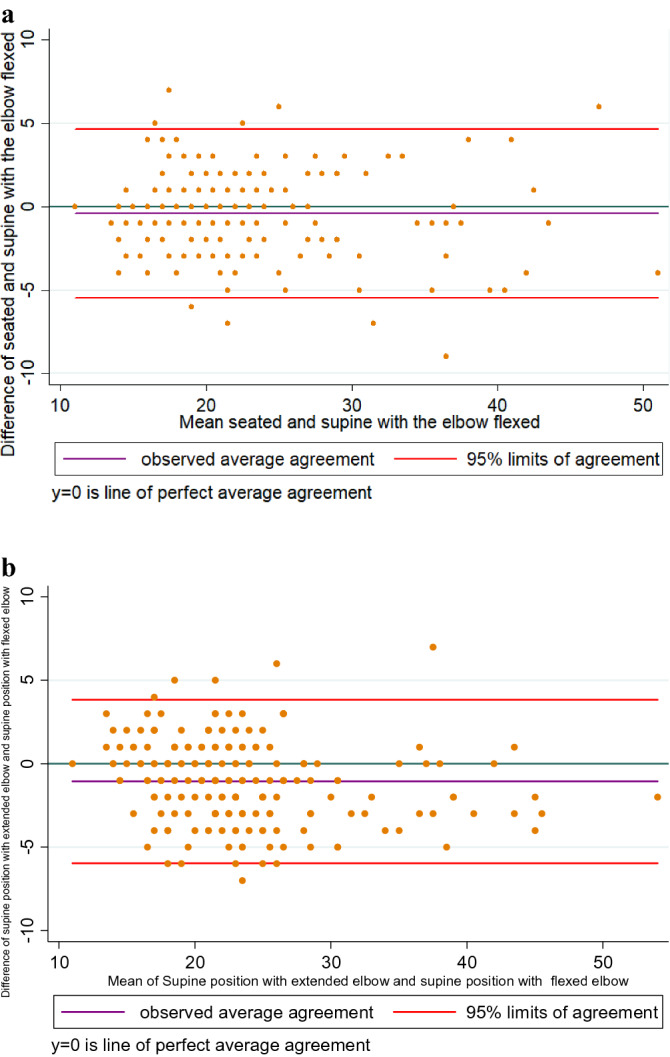
Level of agreement between (**a**) sitting versus supine position with a flexed elbow, (**b**) supine position with an extended elbow and supine position with a flexed elbow.

When comparing the average HS between the right and the left side of each one of the three positions, it was found that the muscular strength of the dominant limb was higher than non-dominant (Table 1). Reproducibility was nearly perfect in all the positions evaluated for both limbs (Table 2). Regarding the level of agreement for the comparison between the sitting and supine positions with a flexed elbow, an average difference of − 0.406 was noted, and the upper and lower agreement limits were found to be 4.592 and − 5.404, respectively. For supine position with an extended and flexed elbow, the average difference was − 1.479, and the upper and lower agreement limits were 3.881 and − 6.840, respectively (Fig. 1).

**Table 2:** Reproducibility test, the reassessment of prehensile strength in three positions.

	Sitting vs. supine with an extended elbow^a^	Sitting vs. supine with a flexed elbow^a^	Supine with a flexed elbow vs. supine with an extended elbow^a^	Supine with a flexed elbow vs. supine with an extended elbow vs. sitting^a^
Right upper limb	0.95 (0.89–0.97)	0.96 (0.95–0.97)	0.95 (0.89–0.97)	0.97 (0.95–0.98)
Left upper limb	0.95 (0.92–0.96)	0.95 (0.94–0.96)	0.95 (0.92–0.96)	0.97 (0.96–0.97)

^a^Data presented as an intraclass correlation coefficient (lower limit and upper limit of the confidence interval).

## Discussion

The findings of this study show that there is agreement between the HGD measured in the supine or sitting position with the elbow flexed or extended. The position recommended by the American Society of Hand Therapists for assessing hand grip dynamometry is a sitting position, with the shoulder in neutral position, the elbow flexed at 90°, and the forearm and wrist in neutral^19^. Most studies perform hand grip dynamometry evaluation in this position^22,23^; however, this position is not always possible to adopt when the measurement is taken at the hospital level, due to situations associated with the patient’s health condition.

The posture of the body and the position of the shoulder, elbow and wrist, influence HS^18,24^. This study evaluated HGD agreement between sitting and supine positions with a flexed and extended elbow. Results showed no significant differences in the measurements between these positions, which suggests that for patients who cannot adopt a sitting posture, measurement taken in the supine position will be equally reliable in determining the level of HGD. In other words, both positions can be interchangeably used since they produce a similar result.

In this study, no differences were noted between HGD values when taking measurements in the sitting or the supine position with a flexed elbow (see Table 1). Although Murugan et al. and Elsais et al., reported a higher value of strength in the sitting position, it was not statistically significant compared with the supine or standing positions^24,25^. These findings can be explained by strength variations associated with the planes in which the movement is performed and to the effect of gravity on the segments involved.

Regarding the position of the flexed elbow at 90°, compared with the extended elbow in the supine position, this study showed a greater HGD value with the elbow in extension (see Table 1). These results are consistent with those reported by España-Romero et al.^18^ and Limbasiya et al.^26^. This can be explained by the fact that an extended position has a more favorable length–tension relationship for the forearm muscles. According to the biomechanical analysis, the flexor digitorum superficialis is the only finger flexor muscle that crosses the elbow joint, which means that the position of this joint can affect the muscle strength developed. As the elbow flexes, the muscle proximally shortens and is mechanically disadvantaged, which decreases its ability to generate tension and, therefore, a better contraction strength^2,7^. However, despite showing a greater HS value in the position with the extended elbow, this difference does not affect the results obtained for reproducibility and limits of agreement; thus, these positions may be interchangeable.

In the present study, differences between HS were identified by gender and dominance; 92% of participants showed right-hand dominance. This is consistent with reports from other studies such as the meta-analysis performed by Dodds et al.^23^, in which the most frequently evaluated hand is the right and/or the dominant hand; as reported in literature the dominant hand has a higher HS value, which is associated with the greater use that the person makes of that hand. In addition, the difference between the strength exerted by men and women is also described in literature^23,24^. In this study, a greater number of women were evaluated, who presented a lower HGD value as opposed to the men that were evaluated, regardless of their posture or position.

Relating to the agreement between the three positions, the coefficients were nearly 1, which means that reproducibility is almost perfect; therefore, the positions can be interchangeable. However, the reproducibility coefficient does not provide information on the pounds of strength variability between measurements. The level of agreement primarily describes the average result from differences obtained between the three positions, which is important when deciding which position would bring less variability in a clinical setting.

When comparing the HS obtained in the sitting position with a flexed elbow, the supine position with a flexed elbow and the supine position with an extended elbow, this study found no differences between the values obtained with HGD (Table 1). This finding is reported in literature^24,25,28^, considering that the effect of body posture and joint position has a low impact on the HS that is not clinically significant.

If the HS determined in each position is similar or equal, the average difference would be expected to be very close to zero. Therefore, a graphical analysis allows for these differences to be quantified and for the upper and lower limits of these differences to be established (Fig. 1). In the present study, these differences may be determined by a true variance, which may be related to a change in muscle strength in each participant, derived from the change in the position, as well as variability recorded by the evaluator and random error.

According to the graphical analysis performed by Bland and Altman (Fig. 1), it can be shown that the average difference is nearly zero, which indicates that the difference in HGD determined by the three positions is approximately 1.5 lb of strength, which could be considered acceptable for a measurement that can calculate approximately 23 lb of strength. The maximum acceptable difference in HGD between the three positions should be considered a clinical interpretation rather than a statistical one^29^.

Another important aspect of measurement variability lies in the analysis of the limits of agreement, which shows the maximum and minimum ranges of measurement variation, which ranged from 3.8 to − 6.8 between the sitting and supine positions with an extended elbow, and from 4.5 to − 5.4 between the sitting and supine positions with a flexed elbow. These limits show a maximum variability of up to 10 lb of strength when the positions are compared, which means that the measurement between the two positions compared can vary by approximately 10 lb. On the basis of the above-mentioned terms, in a clinical or research scenario, this would mean that minor changes of approximately 10 lb of strength could be solely attributed to chance^29^.

## Study limitations

This study did not determine the reproducibility of the test reassessment. It is suggested that future studies should evaluate the variability for each test, which could be a criterion for choosing one of the three positions in cases where it is possible. In addition, the current study examined healthy patients to test the reproducibility and agreement between the three positions; however, the rationale to test different techniques of hand-grip assessment merits a clinical context, so we recommend future studies to evaluate the psychometrical properties of handgrip strength in patients.

## Conclusions

Taking into account the results achieved in this study, clinicians or researchers can choose any of the positions evaluated herein and obtain reliable results as long as the standardization process is followed. The criterion of choice could be the patient’s condition.

## Data Availability

The datasets used and/or analyzed during the current study are available from the corresponding author on reasonable request.

## Abbreviations

HGD: Hand grip dynamometry
HS: Hand Strength
ICC: Intraclass correlation coefficient
